# Oral immunotherapy and anti-IgE antibody treatment for food allergy

**DOI:** 10.1186/s40413-015-0070-3

**Published:** 2015-07-06

**Authors:** Dale T. Umetsu, Rima Rachid, Lynda C. Schneider

**Affiliations:** Genetech, One DNA Way, MS 453b, South San Francisco, California 94080 USA; Division of Immunology and Allergy, Boston Children’s Hospital, Harvard Medical School, Boston, Massachusetts 02115 USA

**Keywords:** Food allergy, Peanut, Oral immunotherapy, Desensitization, Milk

## Abstract

Food allergy is a major public health problem affecting nearly 10 % of children in most industrialized countries. Unfortunately, there are no effective therapies for food allergy, relegating patients to simply avoid the offending foods and treat reactions that occur on accidental exposure. Recently however, studies suggest that food immunotherapy may provide a promising new approach to food allergy, particularly using the oral form of immunotherapy (OIT). Enthusiasm for this approach though must be tempered because of the significant allergic reactions that often occur with OIT that tends to limit its use to patients with less severe disease. On the other hand, recent studies suggest that concomitant treatment of patients with omalizumab (anti-IgE monoclonal antibody) during the updosing phase of OIT may greatly reduce the allergic reactions associated with OIT, even in high-risk patients. This combined method may provide a novel approach to successfully and rapidly treat a large fraction of patients with high-risk food allergy.

## Introduction

Food allergy is a serious public health problem that affects 4-8 % of children in the US [1, 2]. In Australia, the prevalence of peanut allergy alone is 3 % in young children [3]; in the UK it is 2 % of 8-year-old children. In the US, 5 % of adults are estimated to have food allergy; 1.8 % have peanut allergy. Moreover, the prevalence of food allergy appears to have doubled or even quadrupled over the past 15 years in the US, UK and China [4, 5]. Globally, the number of patients with food allergy is estimated to be around 220-250 million [6].

In this review we will focus on IgE-mediated food allergy; non-IgE mediated reactions, such as celiac disease, eosinophilic esophagitis, lactose intolerance or food poisoning, will not be discussed. In IgE-mediated food allergy, reactions begin when allergen binds to IgE bound to the surface of mast cells or basophils through high-affinity IgE receptors (FcεR1), triggering the rapid release of mediators, generally within minutes, including histamine and leukotrienes that cause the symptoms of allergy.

Unfortunately for patients with food allergy, there are no FDA or EMA approved therapies for food allergy, and the standard of care is allergen avoidance and prompt treatment of allergic reactions when they develop after accidental ingestion. However, even when attempting strict avoidance, each patient on average develops a significant allergic reaction every 1-4 years, due to the fact that the major food allergens are often hidden in prepared foods, or may be present due to cross contamination. As a result, food allergy is currently the most common cause of anaphylaxis seen in emergency rooms across the US, with peanut allergy accounting for 50-60 % of fatal episodes of anaphylaxis [7]. Furthermore, food allergy can be very stressful and debilitating for patients and families, because allergic reactions, including anaphylaxis, occur unpredictably. Maladaptive behaviors and anxiety develop, reducing quality of life (QoL) in food allergic children to a greater degree than in children with rheumatologic disease or in children with insulin-dependent diabetes [8, 9]. Therefore, food allergy represents an important and urgent unmet medical need.

### Novel approaches to the treatment of food allergy

To address this need, food immunotherapy has been investigated as a treatment and potentially disease modifying approach. Immunotherapy has been performed subcutaneously, sublingually, transdermally and orally [10]. However, the subcutaneous approach was abandoned many years ago due to safety concerns [11]; the sublingual and transdermal approaches have both been shown to be safe, but efficacy is limited by a restricted dose capacity, i.e., the amount that can be absorbed through the skin or under the tongue [12]. Oral immunotherapy (OIT) is more effective than the other routes, in part because much larger doses can be administered, but safety has been a major limitation [13]. OIT is performed by administration initially of low oral doses, after which the dose is increased as tolerated. Using this method, OIT has been successful in desensitizing many patients to different foods including egg, milk, peanut and tree nut [10, 14, 15]. The goal of therapy in most cases is to reduce or eliminate the severity of reactions following accidental ingestion, which means tolerating relatively low oral maintenance doses; but occasionally, the goal of OIT has been to tolerate much greater maintenance (dietary) doses of the food. However, there is no consensus regarding the best specific protocol in terms of dosing schedule and timing of the doses, and currently OIT is still considered experimental, due to significant concerns regarding safety and long-term consequences, as discussed below.

Although OIT can be effective in increasing the amount of food that can be tolerated by a food allergic individual, allergic reactions, including anaphylaxis, are frequently observed during the desensitization protocol. For example, >90 % of patients undergoing oral milk OIT develop reactions, and 10-20 % of patients require epinephrine at some point during the desensitization process [16]. A recent meta-analysis of OIT studies suggested that the frequent serious adverse reactions might outweigh the benefits of OIT [17, 18]. Moreover, due to frequent allergic reactions that prevent dose increases, OIT generally takes a median time of 20-60 weeks to reach maintenance doses, and the highest oral dose achieved is often below the target maintenance dose. Importantly, as many as 10-30 % of food allergic patients are refractory to desensitization, particularly in patients with higher initial food-specific IgE levels [19–23]. Given these safety issues, OIT is currently performed primarily in academic centers, and most experts strongly believe that it should NOT be recommended for use in the community [24].

### Oral immunotherapy with omalizumab

To address some of the safety issues associated with OIT, anti-IgE monoclonal antibody (mAb) was proposed several years ago as an adjunct to facilitate OIT by reducing OIT-induced allergic reactions [25]. Several previous studies provided the rationale for such an approach. First, treatment with an anti-IgE mAb called Tnx 901 was shown to increase the threshold dose on peanut challenge eliciting allergic reactions from a mean of 90 mg to 1,400 mg of peanut protein [26]. Two additional studies, using another anti-IgE mAb, omalizumab, increased the threshold dose of peanut eliciting allergic reactions on oral peanut challenge [27, 28]. Although there was no intent in these studies to do desensitization, these results suggested that anti-IgE mAb might facilitate oral desensitization.

The first study to use omalizumab with OIT examined desensitization in patients with high-risk, significant milk allergy [25]. Eleven subjects were recruited (median age of 8 years), with high milk-specific IgE (median 50 kU/L, range of 42-342 kU/L) (median total IgE, 349). Patients were pretreated with omalizumab injections every 2-4 weeks, dosed according to the package insert, except for the 3 children with serum IgE levels >700 kU/L, where the dose was 225 to 300 mg (approximately 0.016 mg/kg/IgE [U/mL]) [25], for nine weeks, at which point desensitization began, at a starting dose of 0.1 mg milk powder. Eleven doses were given on the first day, up to a dose of 1,000 mg of milk powder (cumulative dose 2,000 mg, approximately 1 ounce of milk or 1,000 mg milk protein). 7 of 11 patients tolerated the highest dose on the first day; one patient dropped out after the first day of desensitization. The ten remaining patients received daily doses of milk, with weekly up-dosing over the following weeks, while continuing omalizumab. Nine of the 10 patients reached the top dose of 2,000 mg dose, and the 10^th^ patient, who was delayed due to allergic reactions, reached a top dose of 1,000 mg of milk by the seventh week of desensitization, at which point omalizumab was discontinued. Maintenance daily oral milk continued, and at week 25, a double blind placebo controlled food challenge (DBPCFC) was performed, with a total of >8,000 mg milk protein or >8 ounces of milk. Nine of the 10 patients tolerated this challenge, and the 10^th^ patient tolerated 4 ounces of milk. Following the DBPCFC, many of the patients began eating and enjoying ice cream, pizza and other milk containing foods.

### The peanut OIT study

Based on the success of oral milk desensitization with omalizumab, omalizumab was tested next with OIT in high-risk peanut allergic patients. Thirteen patients (median age 10 years, range (7-15) with a history of high-risk peanut allergy, including histories of severe reactions, were recruited (median peanut-specific IgE) was 229 kU/L, range of 21-617 kU/L, the highest median for any oral peanut desensitization study, as far as we know) (median total IgE, 621 kU/L) [29]. Prior to desensitization, a DBPCFC with peanut was performed and all patients reacted to peanut with a median dose of 50 mg peanut protein (<¼ peanut). The patients were then pretreated with omalizumab (using European dosing guidelines) for a total of 12 wks, at which point desensitization started at 0.2 mg of peanut protein, increasing on the first day to a top dose of 250 mg peanut protein (10 doses, cumulative dose, 445 mg). On the first day of the desensitization, all 13 patients reached the top dose of 250 mg peanut protein (>1 peanut) with minimal or no symptoms. All patients continued on daily oral peanut, and with weekly updosing over the following eight weeks to a top dose of 2,000 mg of peanut protein (about 8-10 peanuts). 12 of the 13 patients reached the top dose of 2,000 mg of peanut flour in a median time of 8 weeks, at which point omalizumab was stopped and daily oral maintenance of peanut was continued. One patient dropped out after reaching the 625 mg dose, due to persistent vomiting. Twelve weeks later, the 12 remaining patients passed a DBPCFC, tolerating 4,000 mg of peanut protein, the equivalent of about 16-20 peanuts. Thus, the 12 patients tolerated 160 to 400 times more peanut than they did before desensitization.

### Safety of peanut OIT with omalizumab

Over the course of the 52 week study, including 6 months of observation after discontinuing omalizumab, reactions were graded using the Bock’s scoring system [30]. On the first day of peanut desensitization on omalizumab, minimal or no allergic symptoms developed as all 13 subjects tolerated 445 mg of peanut protein. Over the next eight weeks while on omalizumab, nine patients (70 % of patients, including the one patient who dropped out) had 47 mild and 2 moderate reactions. Surprisingly, 3 patients (23 % of the subjects) developed no symptoms during the whole desensitization process. Thus, while on omalizumab, no severe reactions occurred. The one subject who dropped out, began hypersalivation and vomiting after reaching the 625 mg dose. Except for this one patient, reactions were easily treated with antihistamines, suggesting that omalizumab can protect against severe allergic reactions during the desensitization process. After the omalizumab was discontinued, two subjects had grade 3 (severe) reactions, all occurring at some point after tolerating the 2,000 mg dose of peanut protein or after passing the final DBPCFC. Most of these reactions were associated with exercise, infection, stress, NSAID ingestion or menstrual periods. Reactions during the maintenance period can thus occur, as has been observed in previous studies of OIT without omalizumab [23]. These reactions may not be surprising, since the median peanut-specific IgE at week 52 in our study, though greatly reduced from the start, was still about 70 kU/L. Nevertheless, all of the reactions responded rapidly to treatment, and a few days after the reactions the patients were later able to re-tolerate the 2,000-4000 mg dose. One patient dropped out after developing 2 separate allergic reactions to peanut, after successfully completing the second DBPCFC. Follow up of these patients is currently ongoing and expected to last at least 3-5 years, in order to evaluate if the effect of this desensitization method is long-lasting and also to determine if sustained unresponsiveness/tolerance can be achieved.

### Limitations of the peanut OIT study

There are a number of limitations to this pilot phase 1/2 safety peanut study. The sample size was small, there was no placebo group, and follow-up is limited, although still on-going. Nevertheless, even without a placebo group, several points are worth mentioning. First, the patients in this omalizumab-peanut OIT study had the highest median peanut-specific IgE level, 229 ku/L, which is important, since other investigators in studies without omalizumab have found that higher food-specific IgE levels are associated with greater allergic reactions and failure of patients to be desensitized [19–23]. So in this study, the high rate of successful desensitization is notable, given the high level of peanut specific IgE, and the median time of eight weeks to reach the highest dose of peanut. In contrast, in two previous oral peanut desensitization studies performed without omalizumab, only 10 of 39 patients tolerated the first day top desensitization 100 mg peanut protein dose [31], and in the second study only 6 of 28 patients tolerated the first day 100 mg dose [32]. On the other hand, in the omalizumab-OIT study, 13 of 13 patients tolerated the 250 mg dose, significantly different from the previous two non-omalizumab studies with a P-value <0.0001 (Fisher’s exact test), even though the dose in the omalizumab study was 2.5 times higher than in the other two studies. Moreover, with omalizumab treatment, patients required a median time of 8 weeks to reach the maintenance dose, whereas in previous studies without omalizumab, patients required a median time of 20-60 weeks to the reach maintenance dosing. These differences, particularly given the much higher peanut-specific IgE levels in the omalizumab study population and the low failure rate, strongly suggest that omalizumab facilitates faster oral peanut desensitization with less apparent adverse symptoms than seen historically in other OIT trials.

The idea that omalizumab might facilitate OIT was recently confirmed by a multicenter study performed examining the role of omalizumab in the treatment of cow’s milk allergy. In this trial of omalizumab versus placebo combined with milk OIT, the investigators treated 28 patients with omalizumab and 29 with placebo, and demonstrated that omalizumab significantly reduced dosing symptoms and OIT-related side effects, including a reduction in treatments with epinephrine, from 17 doses in the placebo group to 1 in the omalizumab-treated group (Kim et al. Abstract L19, AAAAI meeting, San Diego, CA 2014). Omalizumab also reduced the time to achieve maintenance dosing (from a median of 31 wks to 26 wks).

Additional support for the effectiveness of omalizumab-facilitated OIT has been provided by two additional publications. The first of these examined OIT to multiple foods after treatment with omalizumab in subjects with multiple food allergies [33]. In this study, 25 patients who had failed an initial double-blind placebo-controlled food challenge at protein doses of 100 mg or less were treated with omalizumab followed by OIT to up to five foods (median 4 foods) simultaneously. 22 of the subjects safely reached and maintained the 4,000 mg doses of each food (median time of 18 wks). A second report described three patients with high-risk egg allergy, two of whom failed conventional egg OIT after the 8.5 mg dose of egg protein, and the third who was thought likely to be refractory to egg OIT because of an ovomucoid-specific IgE of 340 kU/L. All three were successfully desensitized to egg after receiving omalizumab [34]. These results together suggest that omalizumab can facilitate safe OIT, even in patients who might be refractory to conventional OIT.

## Summary

In summary, among children with significant milk allergy or peanut allergy, treatment with omalizumab facilitated rapid oral desensitization, taking as little as eight weeks time to achieve daily maintenance with high doses of the food. Additional follow up of the treated patients however, is required to assess the long-term benefits of desensitization. After reaching maintenance dosing and passing the final DBPCFC, patients occasionally experienced allergic reactions, which were often associated with exercise, infection, stress, menstruation, and NSAID use. The duration and frequency of maintenance therapy must still be worked out. Furthermore, whether sustained unresponsiveness or immunological tolerance develops is not yet clear, although it is likely that maintenance dosing for much longer periods of time will be required to maintain food allergen unresponsiveness. In any case, additional, double-blind placebo-controlled studies are required to further confirm the initial results. Indeed, a placebo controlled study of omalizumab in patients with significant peanut allergy is ongoing, called PRROTECT (Peanut Reactivity Reduced by Oral Tolerance in an anti-IgE Clinical Trial), occurring at 4 sites: Boston Children’s Hospital, Stanford University, Children’s Hospital of Philadelphia, and Lurie Children’s Hospital in Chicago. If this study and other studies replicate the initial four studies, and if the beneficial effects omalizumab-facilitated desensitization persist over time, then a new treatment paradigm, using omalizumab with OIT, could improve the clinical approach for patients with high-risk food allergy.
